# Rosiglitazone Improves Survival and Hastens Recovery from Pancreatic Inflammation in Obese Mice

**DOI:** 10.1371/journal.pone.0040944

**Published:** 2012-07-16

**Authors:** Maria Pini, Davina H. Rhodes, Karla J. Castellanos, Robert J. Cabay, Eileen F. Grady, Giamila Fantuzzi

**Affiliations:** 1 Department of Kinesiology and Nutrition, University of Illinois at Chicago, Chicago, Illinois, United States of America; 2 Department of Pathology, University of Illinois at Chicago, Chicago, Illinois, United States of America; 3 Department of Surgery, University of California San Francisco, San Francisco, California, United States of America; State University of Rio de Janeiro, Biomedical Center, Institute of Biology, Brazil

## Abstract

Obesity increases severity of acute pancreatitis (AP) by unclear mechanisms. We investigated the effect of the PPAR-gamma agonist rosiglitazone (RGZ, 0.01% in the diet) on severity of AP induced by administration of IL-12+ IL-18 in male C57BL6 mice fed a low fat (LFD) or high fat diet (HFD), under the hypothesis that RGZ would reduce disease severity in HFD-fed obese animals. In both LFD and HFD mice without AP, RGZ significantly increased body weight and % fat mass, with significant upregulation of adiponectin and suppression of erythropoiesis. In HFD mice with AP, RGZ significantly increased survival and hastened recovery from pancreatic inflammation, as evaluated by significantly improved pancreatic histology, reduced saponification of visceral adipose tissue and less severe suppression of erythropoiesis at Day 7 post-AP. This was associated with significantly lower circulating and pancreas-associated levels of IL-6, Galectin-3, osteopontin and TIMP-1 in HFD + RGZ mice, particularly at Day 7 post-AP. In LFD mice with AP, RGZ significantly worsened the degree of intrapancreatic acinar and fat necrosis as well as visceral fat saponification, without affecting other parameters of disease severity or inflammation. Induction of AP lead to major suppression of adiponectin levels at Day 7 in both HFD and HFD + RGZ mice. In conclusion, RGZ prevents development of severe AP in obese mice even though it significantly increases adiposity, indicating that obesity can be dissociated from AP severity by improving the metabolic and inflammatory milieu. However, RGZ worsens selective parameters of AP severity in LFD mice.

## Introduction

Acute pancreatitis (AP) is an inflammatory disorder of the exocrine pancreas that results in severe complications, such as multiple organ failure and death, in a subset of patients [1], [2]. Obesity is associated with increased risk of development of severe AP through unclear mechanisms that may include augmented necrosis of intra- and peri-pancreatic fat, fatty pancreas, alterations in the network of cytokines and adipokines, metabolic dysregulation, and reduced respiratory excursion [2]–[7]. Identification of the mechanisms leading to increased risk of severe AP in obese subjects may improve clinical care, thus reducing morbidity and mortality in this population.

Thiazolidinediones (TZDs) are widely used anti-diabetic drugs that act as agonists of the nuclear receptor PPAR-gamma [8]. In addition to regulating insulin sensitivity and lipid partitioning, TZDs are potent inducers of adiponectin, an adipokine with beneficial effects on glucose and lipid metabolism and a modulator of inflammatory responses [9]. Furthermore, activation of PPAR-gamma exerts anti-inflammatory effects in a variety of conditions and experimental models, including AP and pancreatic cancer [8], [10], [11]. Specifically, administration of the TZDs rosiglitazone (RGZ) or pioglitazone exerts beneficial effects in several models of AP, including AP induced by cerulein in mice [12]–[14], sodium taurocholate and L-arginine in rats [15]–[18] as well as post-endoscopic retrograde cholangiopancreatography in rats [19]. However, the effect of RGZ or other TZDs on severe AP in the context of obesity has not been reported. Evaluating the effect of TZDs in the severe AP of obesity is important for several reasons, including: 1) TZDs are commonly used in patients with Type 2 diabetes, who are often overweight or obese [20]; 2) TZDs increase adiposity, which may influence AP severity irrespective of other activities of these drugs [2], [3], [7]; 3) RGZ (and perhaps other TZDs) aggravates development of fatty pancreas in obese mice [21], an effect that may exacerbate AP severity [7], [22].

In the present report, we used the IL-12+ IL-18 murine model of AP to investigate the effect of RGZ in modulating severity of pancreatitis in mice fed a low fat diet (LFD) or high fat diet (HFD). In the IL-12+ IL-18 model, both diet-induced and genetic obesity significantly increase AP severity and delay resolution through mechanisms that include altered inflammatory responses and induction of fat necrosis [4], [5], [7], [23]. Our present data demonstrate that RGZ prevents development of severe AP in HFD-fed obese mice, indicating that obesity can be dissociated from AP severity. However, RGZ does not significantly improve AP severity in LFD lean mice and actually increases degree of acinar and fat necrosis in this group.

## Results

### Effect of RGZ in LFD and HFD Control Mice

To characterize whether RGZ modified the underlying metabolic, hematopoietic and inflammatory milieu before induction of AP, we studied mice receiving LFD or HFD with and without RGZ in the absence of any further treatment. As expected, feeding a HFD lead to a significant increase in body weight, fat mass as well as circulating leptin and insulin levels (Table 1). Administration of RGZ significantly increased body weight and fat mass in both LFD and HFD groups, without significant changes in food intake. There was an 11% increase in body weight and a 73% increase in fat mass in LFD + RGZ *versus* LFD mice, with a 9% increase in body weight and a 23% increase in fat mass observed in HFD + RGZ *versus* HFD mice (Table 1). Circulating levels of adiponectin were 3- and 2-fold higher in RGZ-treated LFD and HFD mice, respectively, compared with the corresponding groups without RGZ. Leptin levels were not significantly altered by RGZ despite the increase in fat mass, indicating a dissociation between adiposity and production of leptin.

**Table 1:** Effect of RGZ in control mice.

	LFD	LFD + RGZ	HFD	HFD + RGZ
Body weight (g)	29.48+1.01	32.80+/−1.79^b^	46.39+/1.04^a^	50.66+/−1.46^ab^
% fat mass	8.57+/−1.42	14.87+/−1.67^b^	23.56+/−0.92^a^	28.94+/−1.40^ab^
Food intake (g/mouse/day)	3.11+/−0.15	3.36+/−0.22	3.50+/−0.30	3.50+/−0.24
Leptin (ng/ml)	2.23+/−0.51	3.07+/−0.76	43.15+/−7.52^a^	39.28+/−7.53^a^
Adiponectin (µg/ml)	7.83+/−1.11	21.02+/−4.91^c^	7.43+/−0.89	15.20+/−8.67^c^
Glucose (mg/dl)	205.6+/−31.0	216.0+/−15.5	257.4+/−25.7	252.6+/−24.1
Insulin (ng/ml)	0.98+/−0.06	0.77+/−0.17	5.80+/−0.50^a^	4.56+/−0.63^ab^
TG (mg/dl)	54.91+/−9.23	47.96+/−13.07	77.33+/−14.20	70.74+/−7.63
Pancreas weight (mg)	172.5+/−13.4	190.4+/−3.9	208.2+/−18.7	213.8+/−23.0
Amylase (U/L)	270.6+/−22.5	296.1+/−29.6	353.4+/−47.9	327.9+/−32.5

Body and pancreas weight, % fat mass, average daily food intake during the last week of feeding with the various diets, plasma levels of leptin, adiponectin, glucose, insulin, triglycerides (TG) and amylase, were measured in LFD and HFD groups with or without RGZ. Data are mean +/− SEM of 6–8 mice per group.

a p<0.001 *vs.* LFD groups;

b p<0.05,

c p<0.001 *vs.* respective group without RGZ by ANOVA.

Addition of RGZ to the diet significantly blunted upregulation of insulin levels in the HFD group, without significant alterations in glucose and triglyceride levels. There was a non-significant trend towards higher weight of the pancreas and elevated plasma amylase levels in HFD groups, irrespective of RGZ administration (Table 1) [24].

In agreement with clinical data [25], [26], RGZ suppressed erythropoiesis in both LFD and HFD mice (Table 2). Reduction in erythrocyte counts and hemoglobin levels by RGZ was accompanied by appropriate suppression of hepcidin expression in the liver (34 and 46% reduction of hepatic hepcidin-1 mRNA levels in LFD and HFD mice receiving RGZ, respectively, compared to mice without RGZ as evaluated by qRT-PCR). As previously reported [27], HFD induced significant leukocytosis, which was prevented by RGZ (Table 2).

**Table 2:** Effect of RGZ on hematological parameters in control mice.

	LFD	LFD + RGZ	HFD	HFD + RGZ
WBC (10^3^/µl)	7.67+/−0.48	5.99+/−0.57	9.18+-0.58 a	7.06+/−1.14
# NE (10^3^/µl)	2.02+/−0.22	1.45+/−0.18	2.19+/−0.21	1.58+/−0.22
# LY (10^3^/µl)	5.92+/−0.52	4.19+−0.40	6.53+/−0.51	5.18+/−0.95
# MO (10^3^/µl)	0.22+/−0.04	0.19+/−0.04	0.27+/−0.03	0.17+/−0.02
# EO (10^3^/µl)	0.11+/−0.04	0.12+/−0.06	0.14+/−0.05	0.10+/−0.04
# BA (10^3^/µl)	0.03+/−0.01	0.05+/−0.03	0.06+−0.02	0.03+−0.01
%NE	24.33+/−1.47	24.05+/−1.27	24.01+/−1.97	22.83+/−0.93
%LY	71.54+/−1.68	70.23+/−2.70	70.83+−2.89	72.14+/−1.89
%MO	2.55+/−0.26	2.99+/−0.47	3.02+/−0.35	2.64+−0.40
%EO	1.21+/−0.34	1.95+−0.74	1.54+−0.52	1.77+/−0.65
%BA	0.37+/−0.13	0.78+/−0.35	0.61+/−0.20	0.61+/−0.21
RBC (10^6^/µl)	9.66+/−0.16	8.79+/−0.19 b	10.00+/−0.06	8.84+/−0.26^b^
Hgb (g/dl)	14.04+/−0.24	12.46+/−0.26 b	14.35+/−0.15	12.30+/−0.34^b^
Hct	45.28+/−1.31	39.8+/−1.04 b	46.08+/−0.71	39.40+/−1.28^b^
MCV (fl)	46.86+/−0.92	45.29+/−0.67	46.10+/−0.52	44.57+/−0.62
MCH (pg/cell)	14.55+/−0.20	14.16+/−0.13	14.35+/−0.10	13.93+/−0.17
Platelets (10^3^/µl)	707+/−65	604+/−33	599+/−72	539+/−36
MPV (fl)	4.73+/−0.10	4.98+/−0.08	4.70+/−0.15	5.09+/−1.05

White blood cell (WBC) counts, Neutrophil (NE), Lymphocyte (LY), Monocyte (MO), Eosinophil (EO) and Basophil (BA) absolute numbers and percentages, Red Blood cell (RBC) numbers, Hemoglobin (Hgb) levels, Hematocrit (Hct), Mean corpuscolar volume (MCV), Mean corpuscolar hemoglobin (MCH), platelet number and Mean Platelet Volume (MPV) were evaluated on EDTA-anticoagulated peripheral blood using a Hemavet 950FS. Data are mean +/− SEM of 6–8 mice per group.

a p<0.05 vs. each other group;

b p<0.001 vs. respective group without RGZ by ANOVA.

**Figure 1:**
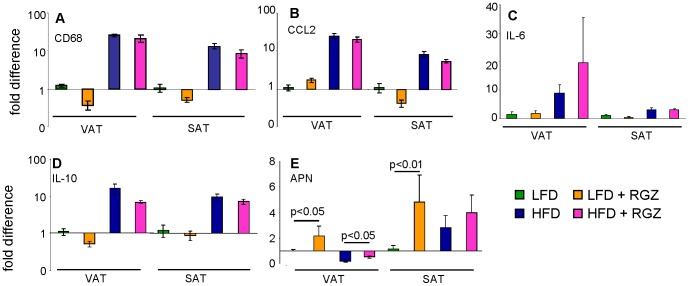
Effect of RGZ on adipose tissue inflammation in LFD and HFD mice. Expression of mRNA for CD68 (**A**), CCL2 (**B**), IL-6 (**C**), IL-10 (**D**), and adiponectin (APN) (**E**) in VAT and SAT were evaluated in mice receiving LFD (green columns), LFD + RGZ (orange columns), HFD (blue columns) or HFD + RGZ (pink columns). Results are expressed as fold increase over VAT or SAT of the LFD group after normalization for expression of housekeeping genes. Data are mean +/− SEM of 5 mice per group.

As expected, expression of the markers of inflammation CD68, CCL2, IL-6 and IL-10 was significantly elevated (p<0.01) in HFD *versus* LFD groups in both visceral (VAT) and subcutaneous (SAT) adipose tissue (Fig. 1 A–D), whereas expression of adiponectin in VAT was significantly lower (p<0.05) in HFD *versus* LFD mice (Fig. 1 E). Administration of RGZ did not significantly alter expression of markers of inflammation in VAT and SAT of either LFD or HFD groups (Fig. 1A–D). However, in LFD mice RGZ significantly increased expression of adiponectin in VAT and SAT (Fig. 1E), in agreement with circulating levels reported in Table 1. Rosiglitazone also blunted the suppression of adiponectin expression induced by HFD in VAT, without significantly altering the elevation of adiponectin mRNA levels induced by HFD in SAT (Fig. 1E).

In summary, RGZ significantly increased adiposity and upregulated expression of adiponectin in adipose tissue. This was associated with amelioration of the metabolic alterations induced by HFD without significant improvement of adipose tissue inflammation. Moreover, RGZ significantly suppressed erythropoiesis in both LFD and HFD mice and prevented development of leukocytosis in the HFD group.

### Effect of RGZ on AP Severity in LFD and HFD Mice

**Figure 2:**
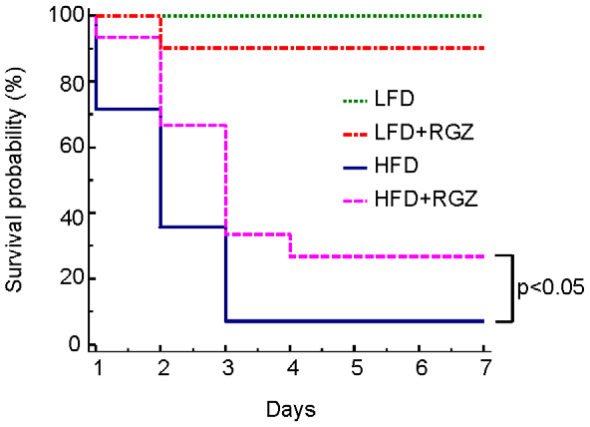
Effect of RGZ on survival from AP in LFD and HFD mice. Mice in the LFD (green line), LFD + RGZ (orange line), HFD (blue line) or HFD + RGZ (pink line) received two injections of IL-12+ IL-18 at 150 and 750 ng/mouse each, respectively and survival monitored for 15 days. No further lethality was observed after Day 7. Data are from 10–15 mice per group.

Administration of IL-12+ IL-18 induces lethality selectively in obese mice, with 100% survival in lean animals [4], [5], [7]. To investigate whether RGZ affected survival in this model of pancreatitis, LFD, LFD + RGZ, HFD and HFD + RGZ mice were injected with a high dose of IL-12+ IL-18 and monitored for 15 days. As shown in Fig. 2, 100% survival was observed in LFD mice, while LFD + RGZ had 90% survival. However, the difference between the two LFD groups did not reach statistical significance. In contrast, significantly increased lethality was observed in both HFD and HFD + RGZ mice compared to lean animals (p<0.001 by Kaplan-Meyer analysis). Presence of RGZ in the diet significantly improved the survival rate of HFD mice (27% vs. 7% survival in HFD + RGZ *versus* HFD, respectively, p<0.05, Fig. 2). No further lethality was observed after 7 days (not shown).

**Figure 3:**
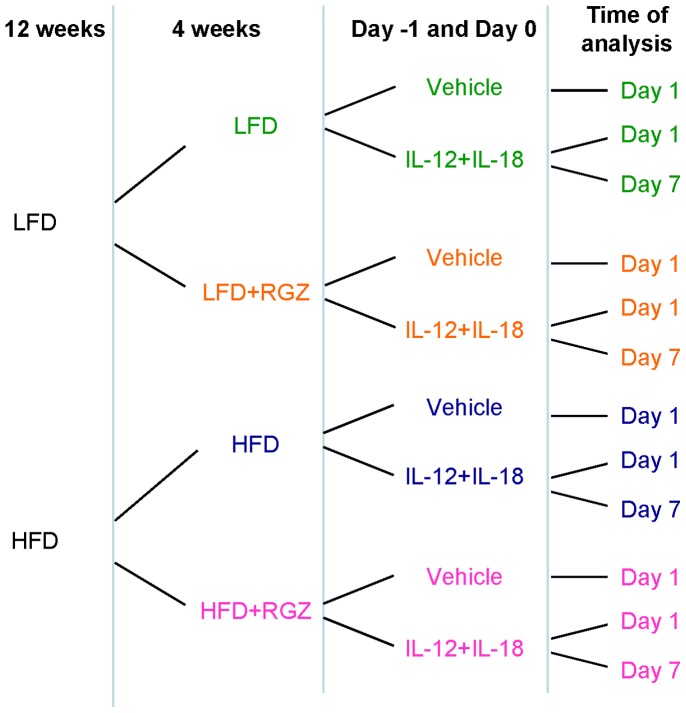
Experimental design. Timing of LFD and HFD feeding with and without RGZ, administration of vehicle or IL-12+ IL-18 (2 injections, 24 h apart), and termination of the experiment is shown.

**Figure 4:**
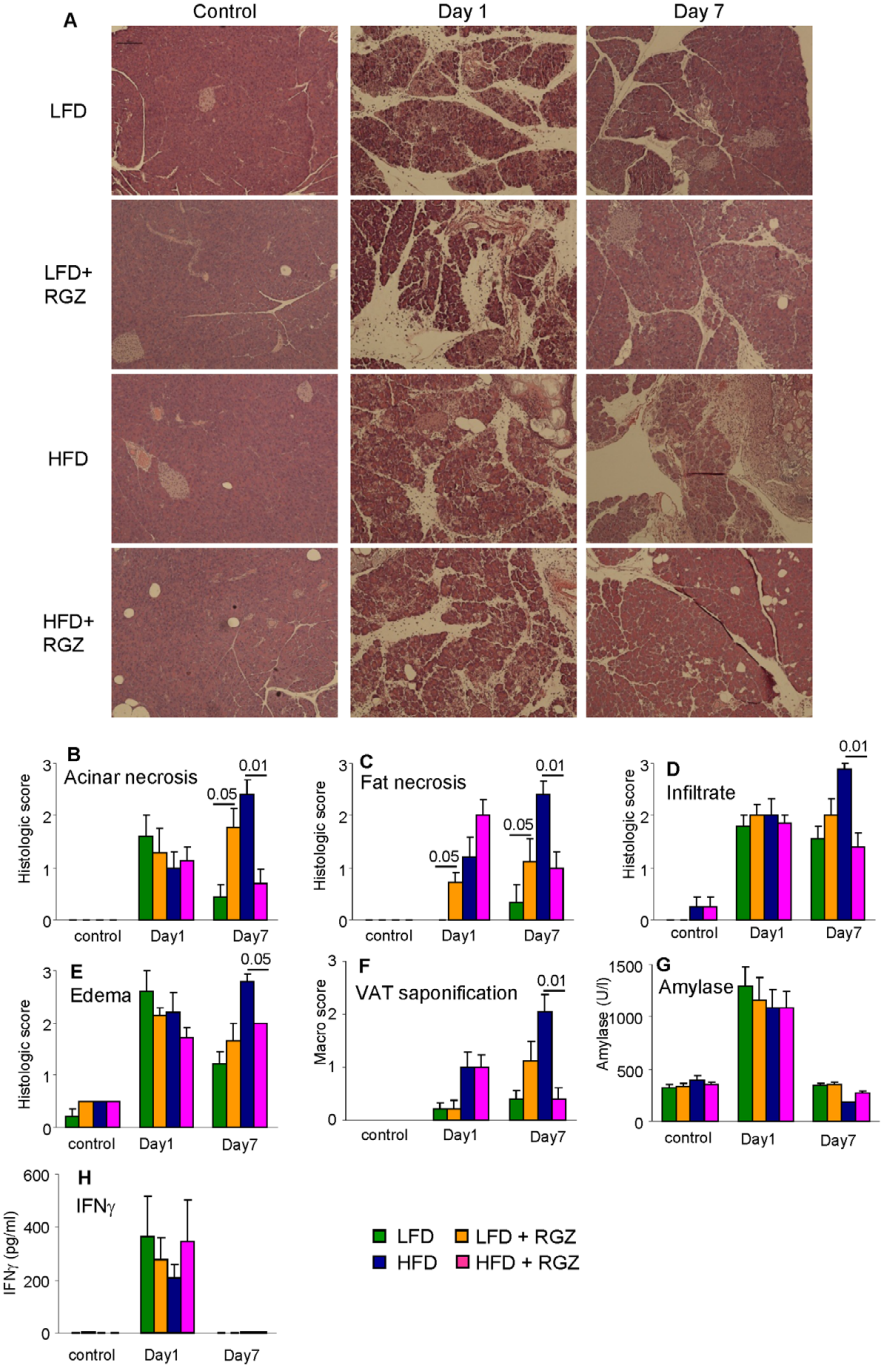
Effect of RGZ on AP induced by IL-12+ IL-18. Mice in the LFD (green columns), LFD + RGZ (orange columns), HFD (blue columns) or HFD + RGZ (pink columns) groups received two injections of IL-12+ IL-18 and were evaluated at Day 1 and Day 7. Control mice received vehicle. **R**epresentative H&E staining of sections of the pancreas as shown in Panel A. Histological scores for pancreatic acinar necrosis (**B**), fat necrosis (**C**), inflammatory infiltrate (**D**) and edema (**E**) as well as VAT saponification (**F)** were calculated as described in the Methods section. Levels of amylase (**G**) and IFN-gamma (**H**) were measured in plasma. Data are mean +/− SEM of 8–12 mice per group.

In order to evaluate the effect of RGZ on the recovery phase of AP, we used a lower dose of IL-12+ IL-18 to avoid lethality. Mice were evaluated at Day 1 and Day 7 after the second injection (Fig. 3). Administration of the cytokine combination at this dose induced pancreatitis in each group, as demonstrated by development of pancreatic damage (Fig. 4 A–E) and significant increase in plasma amylase and IFN-gamma levels (p<0.001) at Day 1 (Fig. 4G–H). No lethality was observed at this lower dose of IL-12+ IL-18.

Compared to LFD mice without RGZ, the LFD + RGZ group had a significantly higher score for acinar necrosis at Day 7 (Fig. 4B) and intrapancreatic fat necrosis at both Days 1 and 7 (Fig. 4C), whereas the degree of pancreatic inflammatory infiltrate and edema was comparable in LFD and LFD + RGZ groups at each time point (Fig. 4 D and E). The LFD + RGZ group also had a significantly higher score for VAT saponification compared to LFD mice at Day 7 (Fig. 4F).

We previously demonstrated that mice receiving HFD develop more severe and prolonged AP compared to mice receiving LFD [5], [23]. In agreement with these results, histological scores for acinar and fat necrosis, inflammatory infiltrate and edema were each significantly elevated (p<0.05) in the HFD group compared with LFD mice at Day 7 (Fig. 4 B–E). Presence of RGZ in the HFD did not significantly alter the response at Day 1. However, pancreatic damage in HFD + RGZ mice was beginning to resolve by Day 7, with scores for acinar necrosis and inflammatory infiltrate comparable to those observed in LFD groups, and fat necrosis and edema scores being intermediate between LFD and HFD mice (Fig. 4 B–E). Both HFD and HFD + RGZ mice developed significant and comparable VAT saponification at Day 1. However, the degree of VAT saponification worsened by Day 7 in HFD mice, whereas improvement was observed in HFD + RGZ mice (Fig. 4F).

**Figure 5:**
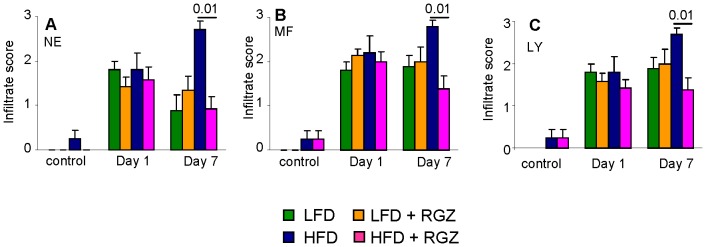
Effect of RGZ on the pancreatic inflammatory infiltrate. Mice received two injections of IL -12+ IL-18 and were evaluated at Day 1 and Day 7. Control mice received vehicle. Infiltration by neutrophils (**A**), macrophages (**B**) and lymphocytes (**C**) was quantified by a pathologist in sections of pancreas obtained from LFD (green columns), LFD + RGZ (orange columns), HFD (blue columns) or HFD + RGZ (pink columns) groups. Data are mean +/− SEM of 8–12 mice per group.

Characterization of the pancreatic leukocytic infiltrate of HFD and HFD + RGZ mice indicated that RGZ hastened resolution of the neutrophil, macrophages and lymphocyte infiltrate, without selective effects on specific cell types (Fig. 5).

In summary, RGZ improved survival and hastened recovery from pancreatitis in HFD mice, leading to an overall response comparable to that of the LFD group at Day 7. However, administration of RGZ worsened selective parameters of disease severity in LFD mice.

### Effect of RGZ on Pancreatic and Circulating Inflammatory Mediators in Mice with AP

**Figure 6:**
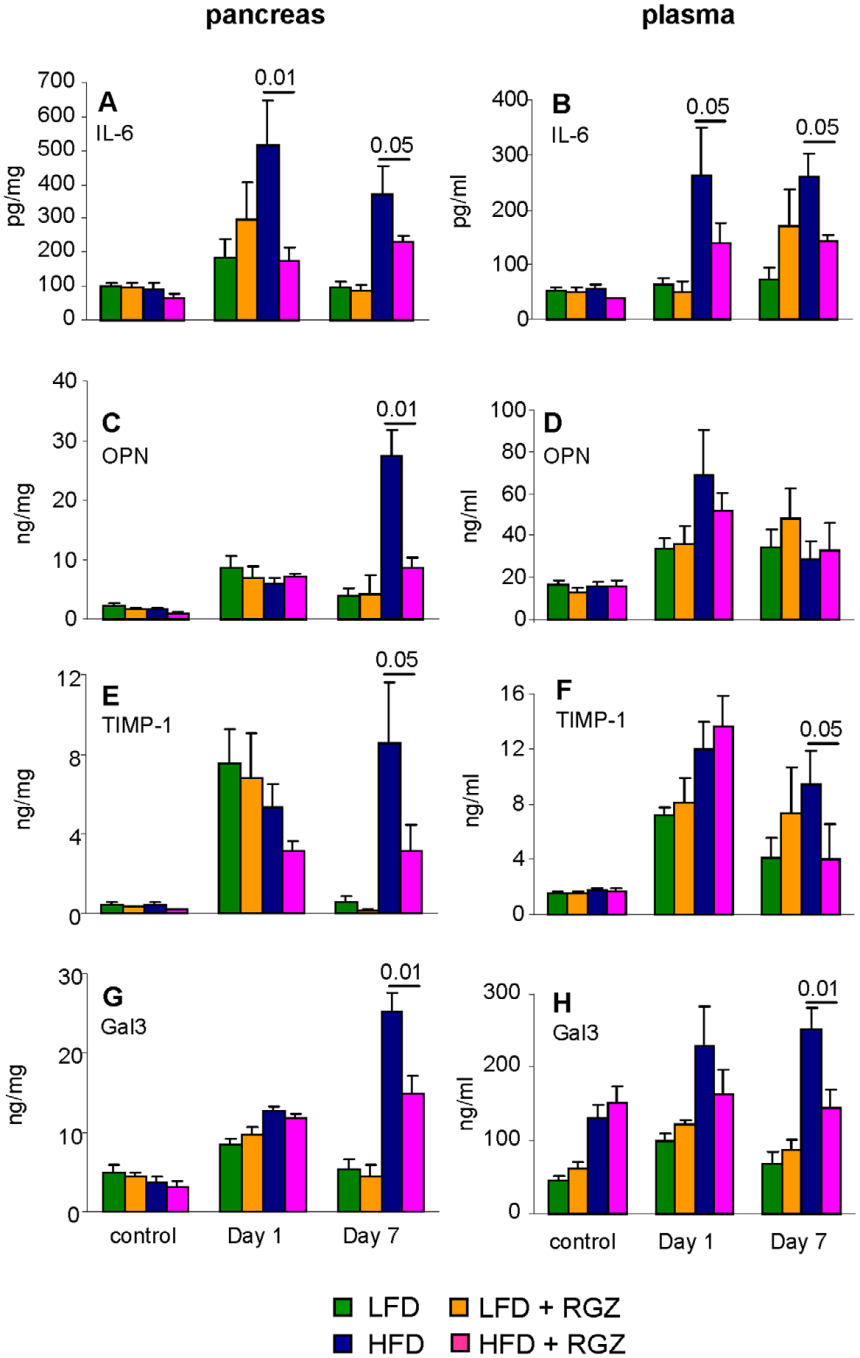
Effect of RGZ on pancreatic and circulating inflammatory mediators. Mice received two injections of IL -12+ IL-18 and were evaluated at Day 1 and Day 7. Control mice received vehicle. Levels of by IL-6 (**A–B**), osteopontin (OPN) (**C–D**), TIMP1 (**E–F**) and Galectin-3 (Gal3) (**G–H**) were quantified in pancreatic homogenates (**A, C, E, G**) and plasma (**B, D, F, H**) obtained from LFD (green columns), LFD + RGZ (orange columns), HFD (blue columns) or HFD + RGZ (pink columns) groups. Data are mean +/− SEM of 8–12 mice per group.

We recently demonstrated that sustained elevation of IL-6 in obese mice receiving IL-12+ IL-18 contributes to the delayed resolution of AP in obesity and is associated with increased production of osteopontin and tissue inhibitor of metalloproteinase-1 (TIMP-1) [5]. As shown in Fig. 6 A and B, RGZ significantly blunted the upregulation of IL-6 induced by IL -12+ IL-18 in the pancreas and systemic circulation of HFD mice at both Day 1 and Day 7, without affecting the response in LFD mice. Rosiglitazone also significantly reduced pancreatic levels of osteopontin (Fig. 6C) as well as both pancreatic and plasma levels of TIMP-1 (Fig. 6 E–F) at Day 7 in HFD mice, with no significant effects in LFD mice.

Levels of the pro-inflammatory mediator Galectin-3 were significantly elevated (p<0.01) at comparable levels in pancreatic homogenates of each group at Day 1. By Day 7, pancreatic levels of Galectin-3 had returned to baseline in LFD and LFD + RGZ mice, while they were still elevated in HFD and HFD + RGZ mice (Fig. 6G). However, pancreatic Galectin-3 levels at Day 7 were significantly higher in HFD compared to HFD + RGZ mice. Baseline plasma Galectin-3 levels were significantly higher in HFD and HFD + RGZ mice compared to LFD groups (p<0.01), as we recently demonstrated [28] (Fig. 6H). Administration of IL-12+ IL-18 significantly increased plasma Galectin-3 levels in LFD and LFD + RGZ mice at Day 1 (p<0.01), with levels returning to baseline at Day 7. In HFD mice, plasma Galectin-3 was significantly elevated at both Day 1 and Day 7 compared to baseline and to both LFD groups (p<0.001), whereas AP did not significantly alter plasma Galectin-3 levels in the HFD + RGZ group at any time point (Fig. 6H).

In summary, administration of RGZ to HFD mice significantly blunted induction of IL-6 and was associated with patterns of production of osteopontin, TIMP-1 and Galectin-3 similar to those of LFD groups or intermediate between LFD and HFD mice.

### Effect of RGZ on Hematological Parameters in Mice with AP

**Figure 7:**
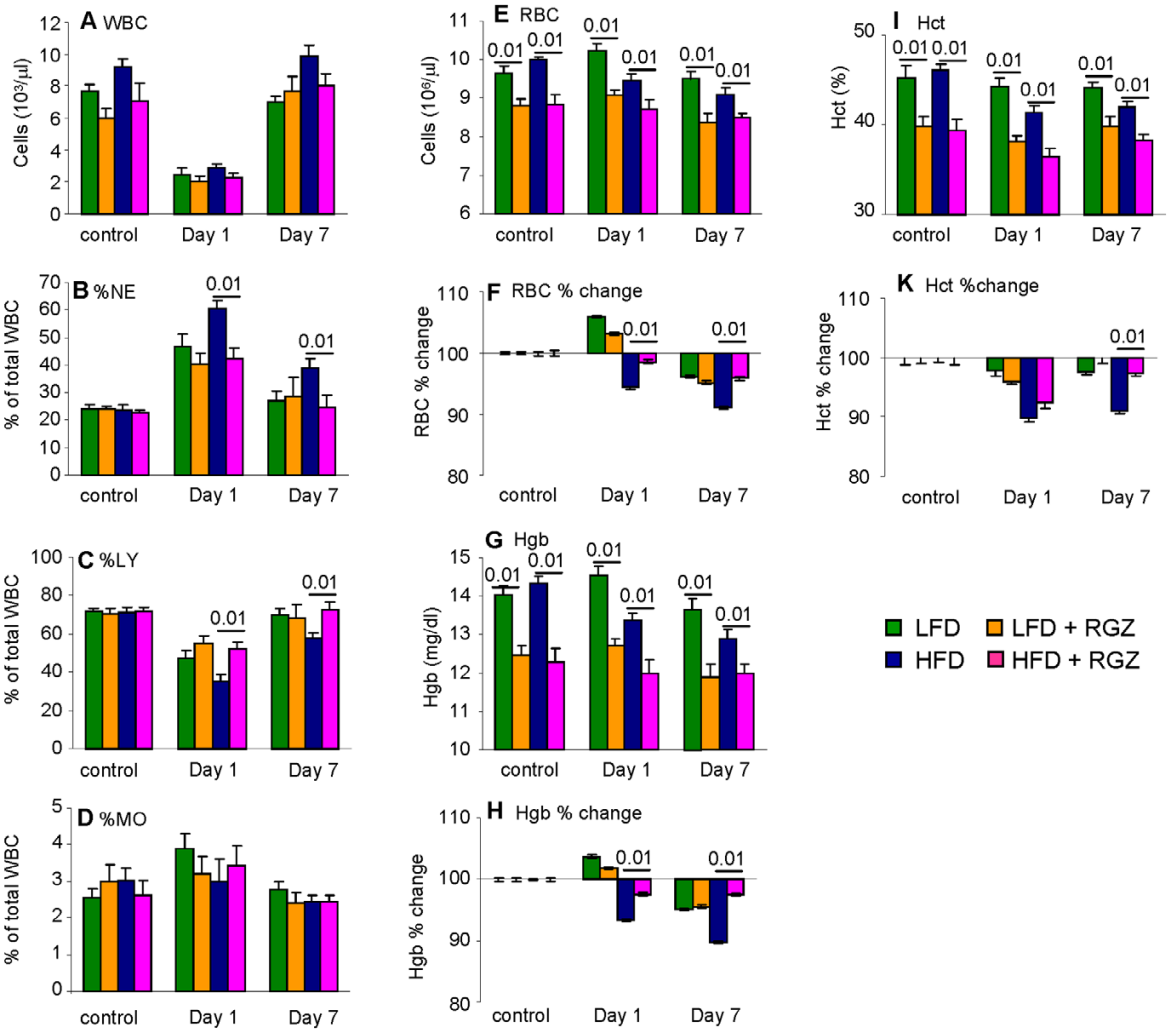
Effect of RGZ on hematological parameters in mice with AP. Mice received two injections of IL -12+ IL-18 and were evaluated at Day 1 and Day 7. Control mice received vehicle. Circulating while blood cells (WBC) (**A**), % neutrophils (**B**), % lymphocytes (**C**) and % monocytes (**D**) as well as red blood cells (RBC) (**E** absolute value, **F** % change from baseline), concentration of hemoglobin (**G** absolute value, **H** % change from baseline), and hematocrit (**I** absolute value, **I** % change from baseline) were quantified in blood obtained from LFD (green columns), LFD + RGZ (orange columns), HFD (blue columns) or HFD + RGZ (pink columns) groups. Data are mean +/− SEM of 8–12 mice per group.

As reported in Table 2 and in agreement with previous results [27], significantly higher numbers of leukocytes (p<0.05) were present in the circulation of HFD mice without AP (control) compared with each other group, with a comparable pattern observed at Day 7 (Fig. 7A). Administration of IL-12+ IL-18 induced significant leukopenia of comparable magnitude in each group at Day 1 (p<0.01), with complete recovery by Day 7 (Fig. 7A). Leukopenia at Day 1 was associated with a significant increase in the percentage of circulating neutrophils (p<0.001) and monocytes (p<0.05) and a decrease in percentage of lymphocytes (p<0.001) in each group (Fig. 7B–D). Although the magnitude of leukopenia was comparable in each group, the percentage of neutrophils was significantly higher and percentage of lymphocytes significantly lower in HFD mice *versus* each other group at both Days 1 and 7, whereas changes in monocytes were comparable in each group (Fig. 7B–D).

Administration of IL-12+ IL-18 also induced alterations in the erythrocyte compartment. To correct for the suppressive effect of RGZ on erythropoiesis (see Table 2) and allow for direct comparison of the magnitude of change induced by IL-12+ IL-18 in the presence and absence of RGZ, data were analyzed as absolute values as well as percent change in erythrocyte counts, hemoglobin and hematocrit levels compared to the respective control groups without AP. As shown in Fig. 7 E–K, administration of IL -12+ IL-18 significantly suppressed erythropoiesis in HFD mice (p<0.05), with only minor alterations observed in LFD groups. The magnitude of inhibition of erythropoiesis was significantly blunted in the HFD + RGZ group compared with HFD mice (Fig. 7 F, H and K). However, as a result of the suppressive effect of RGZ on erythropoiesis, absolute erythrocyte, hemoglobin and hematocrit values were always significantly lower in RGZ groups compared to mice not receiving RGZ (Fig. 7 E, G and I).

To summarize, the hematological response to IL-12+ IL-18 was altered in HFD compared to LFD mice, with RGZ preventing these alterations and leading to a response of the HFD + RGZ group comparable to that observed in LFD mice.

### Effect of RGZ on Adipokine Levels in Mice with AP

**Figure 8:**
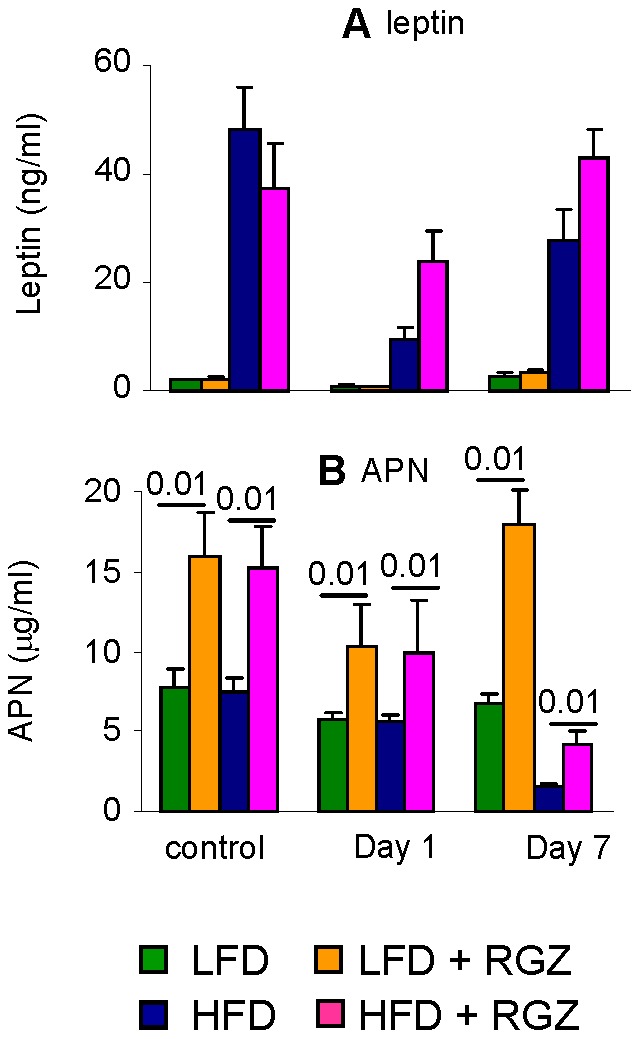
Effect of RGZ on adipokine levels in mice with AP. Mice in the LFD (green columns), LFD + RGZ (orange columns), HFD (blue columns) or HFD + RGZ (pink columns) groups received two injections of IL-12+ IL-18 and were evaluated at Day 1 and Day 7. Control mice received vehicle. Circulating levels of leptin (**A**) and adiponectin (APN) (**B**) were measured by ELISA. Data are mean +/− SEM of 8–12 mice per group.

Administration of IL-12+ IL-18 lead to a significant decrease in plasma leptin levels in all mice at Day 1 (p<0.01), with a return to baseline values by Day 7 in each group except HFD mice (Fig. 8A). Despite the significant decline, plasma leptin levels remained significantly higher in HFD compared to LFD groups at each time point (p<0.001).

No significant changes in adiponectin levels were observed in any group at Day 1 post-AP (Fig. 8B). At Day 7 circulating levels of adiponectin were suppressed by 78% and 73% in HFD and HFD + RGZ mice, respectively, compared to baseline values (p<0.001), with no significant alterations observed in LFD groups. However, because HFD + RGZ mice had higher baseline levels, circulating adiponectin in this group remained significantly higher at Day 7 compared to HFD mice despite the parallel decline (Fig. 8B). Thus, AP leads to major suppression of adiponectin production in HFD mice irrespective of RGZ.

## Discussion

In the present report we demonstrate that RGZ prevents development of severe AP induced by IL-12+ IL-18 in mice fed a HFD, as evaluated by decreased lethality, improved pancreatic histology and hematological alterations as well as reduced induction of inflammatory mediators. However, RGZ did not ameliorate, and actually tended to worsen, AP in LFD groups.

### Effect of RGZ in Control Mice

As previously demonstrated in animal models and humans [8], RGZ significantly increased body weight and fat mass in both LFD and HFD mice. However, increased adiposity was associated with a favorable metabolic profile, as indicated by reduced insulin concentrations and major upregulation of circulating levels and adipose tissue expression of adiponectin, again in agreement with previous results [8]. Despite the demonstrated anti-inflammatory effects of PPAR-gamma activators [8], the favorable metabolic effects of RGZ were not accompanied by amelioration of adipose tissue inflammation induced by HFD and obesity. This is in agreement with previous reports demonstrating lack of effect of RGZ on markers of adipose tissue inflammation, such as TNF-alpha, IL-6, CD68 and CD11c [29], [30] and with the observation that - compared with other TZDs - RGZ is a weak anti-inflammatory agent in adipose tissue of obese rats [31]. However, controversy exists in this field, as other reports indicate that RGZ can effectively prevent VAT inflammation induced by HFD [32]. Nevertheless, under the experimental conditions of our study, induction of adiponectin and reduction of insulin levels by RGZ were dissociated from effects on adipose tissue inflammation.

We also demonstrate that chronic administration of RGZ inhibited erythropoiesis in both LFD and HFD mice, with significant reductions in erythrocyte counts as well as hematocrit and hemoglobin levels. Inhibition of erythropoiesis in mice receiving RGZ was accompanied by appropriate downregulation of hepcidin expression in the liver, suggesting the effect was not secondary to dysregulated iron metabolism [33]. These results are in agreement with clinical data demonstrating reduction of hematocrit and hemoglobin levels in patients treated with TZDs, including RGZ [26], [34]. The exact mechanisms of suppressed erythropoiesis by TZDs remain unclear, with increased adipogenesis in the bone marrow and decreased insulin levels proposed as potential mediators [26], [35]. Our results demonstrating that the suppressive effect of TZDs on erythropoiesis can be reproduced in mice provide an experimental model for identifying the mechanisms of this side-effect of TZDs.

### Effect of RGZ in Mice with AP

Administration of RGZ increased survival, ameliorated pancreatic damage and VAT necrosis, and improved the inflammatory and hematopoietic response to administration of IL-12+ IL-18 in HFD mice. These results are in line with previous reports demonstrating a beneficial effect of RGZ in various experimental models of AP in non-obese mice [12]–[19]. The novelty of our results is the demonstration that RGZ is protective in a model of severe necrotic AP associated with lethality in obese mice [4], [5], [7]. Because TZDs are used as therapeutic agents in Type 2 diabetic patients, who are often overweight/obese, these data may have potential translational significance, although their relevance in humans needs to be confirmed.

However, at variance with the protective effect of RGZ in HFD mice and with previous reports [12]–[19], our data indicate that RGZ worsened acinar and fat necrosis in LFD mice. Increased adiposity induced by RGZ is the most likely mechanism for this finding, since the LFD + RGZ group had a major (73%) increase in fat mass compared with LFD mice not receiving RGZ. Differences in experimental protocols likely explain the discrepancy between our findings and previous studies. In fact, in the majority of previous reports TZDs had been administered acutely before or shortly after induction of AP [12], [14]–[16], [18], [19], thus circumventing the adipose tissue-expanding effect of these drugs. On the other hand, the cerulein model of AP, which does not induce fat necrosis, was used in the single study in which RGZ had been administered chronically before induction of AP [13]. Thus, a fine balance between increasing adiposity and reducing inflammation may determine the effect of TZDs on AP’s outcome.

The protective effect of RGZ in HFD mice was associated with reduced production of IL-6, osteopontin, TIMP-1 and Galectin-3. Elevated levels of IL-6 are one of the best predictors of disease severity in AP [1]. However, although previous studies indicated that elevated production of IL-6 delays recovery from pancreatic damage and mediates induction of osteopontin and TIMP-1, high IL-6 was not responsible for increased lethality of obese mice injected with IL-12+ IL-18 [5]. Furthermore, at variance with the effect of RGZ, IL-6 deficiency selectively reduced the neutrophilic infiltrate in the pancreas of obese mice with AP, without effects on macrophages and lymphocytes [5]. Therefore, suppression of IL-6 and IL-6-induced mediators in HFD mice with AP is likely only part of the protective mechanism of RGZ. We previously demonstrated that neutralization of IFN-gamma protects both lean and obese mice from AP induced by IL-12+ IL-18 [4]. However, RGZ did not significantly alter induction of IFN-gamma, suggesting an alternative way of action. Navina et al. demonstrated that the lipase inhibitor Orlistat prevents induction of fat necrosis and protects *ob/ob* mice from AP-associated lethality induced by IL-12+ IL-18 [7]. Our present data indicate that RGZ hastened recovery from both intrapancreatic fat necrosis and VAT saponification in obese mice, even though it did not affect the acute response at Day 1. These findings suggest that interventions that reduce the extent of fat necrosis in AP are associated with beneficial effects, as supported by evidence of a correlation between peripancreatic fat necrosis and disease severity in human AP [36]. Finally, a potential mechanism by which RGZ might have afforded protection in HFD mice is by elevating production of adiponectin prior to induction of AP. In fact, low levels of adiponectin are associated with organ failure in patients with AP [37]. Furthermore, adiponectin protects HFD-fed mice from cerulein-induced AP and inhibits production of IL-6 in *db/db* mice [38], [39]. Thus, the high baseline levels of adiponectin in HFD + RGZ mice may have contributed to control the excessive elevation of IL-6 of obese mice. However, elevated levels of adiponectin may have antithetical effects depending on the target tissue and specific microenvironmental conditions, as indicated by the pro-inflammatory effects of adiponectin in the joint and the paradoxical increase in circulating adiponectin levels in several chronic inflammatory diseases [9], [40]. Nevertheless, it is important to note that inflammation induced by IL-12+ IL-18 is a very potent suppressor of adiponectin production even in the presence of RGZ.

### Conclusions

Alterations in the metabolic and inflammatory milieu induced by the PPAR-gamma activator RGZ effectively dissociate obesity from severe AP. However, there is a fine balance between the anti-inflammatory and adiposity-inducing effects of RGZ, as indicated by worsening of some parameters of AP in non-obese animals receiving RGZ.

## Materials and Methods

### Ethics Statement

Animal studies were approved by the Animal Care and Use Committee of the University of Illinois at Chicago under protocol A10–008.

### Animals and Diets

Male C57BL6 mice (The Jackson Laboratories, Bar Harbor, ME) were fed a LFD (10% Kcal/fat; 7% Kcal/sucrose) or a HFD (60 Kcal% fat7% Kcal/sucrose, D12492, from Research Diets, New Brunswick, NJ) *ad libitum* for 12 weeks beginning at 4 weeks of age. After 12 weeks of feeding, half of the mice in each group were switched to a LFD or HFD that contained 0.01% RGZ (Research Diets) for 4 weeks resulting in 4 experimental groups: LFD, LFD + RGZ, HFD, HFD + RGZ. The concentration of RGZ was selected based on previously published studied [30].

### Control Groups

A subset of mice in each the four experimental groups was euthanized without further treatment for evaluation of the effect of RGZ on inflammatory, hematologic and metabolic parameters. Body composition was analyzed by Dual-energy X-ray absorptiometry. Food intake was evaluated during the last week of feeding. Epididymal VAT, SAT and liver were immediately frozen in liquid nitrogen and stored at −70°C until processing. Total RNA was isolated using Trizol and reverse transcribed. Gene expression of adiponectin, CCL2, CD68, IL-6, and IL-10 was evaluated in VAT and SAT, whereas expression of hepcidin-1was assessed in liver by real-time RT-PCR using the TaqMan system and primers from Applied Biosystems (Foster City, CA). Relative expression was calculated using the Delta-Delta^CT^ method after normalizing for expression of the geometric mean of GAPDH, actin and S18. Hematologic parameters and circulating adipokine levels were measured as detailed below.

### Induction of AP

Murine recombinant IL-12 and IL-18 (R&D Systems, Minneapolis, MN) were administered i.p. at 24 h intervals, for a total of 2 injections, as previously described [4], [23]. In separate experiments we used the cytokine combination at different concentrations: 150 ng/mouse of IL-12 and 750 ng/mouse of IL-18 for experiments evaluating survival, and 100 ng/mouse of IL-12 and 500 ng/mouse of IL-18 for experiments evaluating resolution of inflammation. Mice were euthanized at either 1 or 7 days after the second injection of IL-12+ IL-18. Groups used as healthy controls for AP received vehicle and were euthanized 1 day after the second injection. Severity of VAT necrosis and saponification was quantified macroscopically as 0 (absent), 1 (few pinhead-sized necrotic areas, without retropancreatic necrosis), 2 (moderately extended necrotic areas with moderate/extensive retropancreatic necrosis), 3 (extensive areas of necrosis with extensive retropancreatic necrosis). Blood was collected at time of euthanasia in EDTA tubes. After evaluation of hematologic parameters using the HV950FS (Drew Scientific, Waterbury, CT), blood was centrifuged and plasma obtained and stored at −70°C for subsequent analysis. The pancreas was obtained and a portion fixed in formalin for histological evaluation, while the remaining tissue was immediately frozen in liquid nitrogen and stored at −70°C for subsequent processing. Scoring for pancreatic damage was performed on hematoxylin/eosin-stained sections by a pathologist (RJC) blinded to the experimental groups using a previously described scoring system [4].

### Miscellaneous Measurements

Levels of IL-6, IFN-gamma, leptin, adiponectin, Galectin-3, osteopontin and TIMP-1 were measured using ELISA kits from R&D Systems (Minneapolis, MN). Serum amylase and triglyceride levels were measured using kits from Teco Diagnostics (Anaheim, CA). Pancreas homogenates were prepared by homogenizing tissue in Cell Lysis buffer (Cell Signaling, Danvers, MA), followed by sonication. Protein concentration was adjusted to 1 mg/ml for measurement of IL-6, Galectin-3, osteopontin and TIMP-1 by ELISA.

### Statistical Analysis

Data are expressed as mean +/− SEM. Statistical significance of differences were determined by one-way ANOVA. The Kaplan-Meyer method was used for analysis of survival data. Statistical analyses were performed using the MedCalc software (Mariakerke, Belgium).
